# Bedside gastrointestinal ultrasound combined with acute gastrointestinal injury score to guide enteral nutrition therapy in critically patients

**DOI:** 10.1186/s12871-022-01772-9

**Published:** 2022-07-19

**Authors:** Jiawei Lai, Shuhong Chen, Linli Chen, Daofeng Huang, Jinzhan Lin, Qingjiang Zheng

**Affiliations:** Department of Critical Care Medicine, Zhangzhou Affiliated Hospital of Fujian Medical University, No 59, Shengli West Road, Zhangzhou, 363000 Fujian China

**Keywords:** Distal gastric ultrasound, Colon ultrasonic, Enteral nutrition therapy

## Abstract

**Background:**

To use gastric ultrasound to evaluate function and to determine the start time of enteral nutrition (EN) in patients with acute gastrointestinal injury (AGI).

**Methods:**

We reviewed records from 105 patients who suffered AGI levels two (AGI II). We recorded several data points, including ultrasonographic transverse area of gastric antrum (CSA), left descending colonic or right ascending colonic diameter (Diam), peristatic frequency (Peri), EN start time, EN dose, prealbumin (PA), and EN complications. The recovery of intestinal function after EN treatment was judged as success. If there was EN treatment complication, this was judged as failure. We analyzed the changes in gastrointestinal function after EN treatment, to determine feeding time.

**Results:**

There were 69 patients in the successful group, and 36 in the failure group. There were no significant differences between the two groups in age, intra abdominal pressure (IAP), APACHE II, PA and disease composition (*p* > 0.05).There were significant differences in terms of EN startup time, CSA, Diam, Peri, and PA, between the EN success and failure groups. We found IAP does not reflect gastrointestinal function;CSA ≤ 9cm2, Diam ≤ 2.9 cm, Peri > 3 bpm, indicated that the three indexes could reflect the recovery of gastrointestinal function. Receiver operating curve analysis showed that combined CSA, Diam, Peri evaluation determined the best time to start EN.

**Conclusions:**

Monitoring gastric antrum transversal area, colonic diameter, colonic peristatic frequency using ultrasound can guide the timing of initiation of enteral nutrition treatment.

**Supplementary Information:**

The online version contains supplementary material available at 10.1186/s12871-022-01772-9.

## Introduction

When the body suffers serious damage, it is easy to cause spasmodic contraction of the gastric and mesenteric arteries, and then lead to gastrointestinal mucosal ischemia necrosis. This in turn leads to acute gastrointestinal injury (AGI)., manifesting as gastrointestinal peristalsis, absorptive dysfunction, gastrointestinal bleeding, intestinal dilatation, and other complications.

When the patient’s condition is stabilized, early enteral feeding initiation is recommended, followed by transition to targeted feeding. Enteral nutrition therapy for severely ill patients can promote the recovery of gastrointestinal function, maintain mucosal integrity, and correct intestinal flora imbalance.. Currently, it is recommended to start EN within 24 to 48 h after the patient is stabilized, and to continue feeding for 24 h [1, 2]. The AGI score is based on subjective assessment (the only objective indicator is IAP).Severely ill patients are often unable to cooperate to the point of generating the score properly, either because of invasive mechanical ventilation, need for analgesia and sedation, or many other reasons. Therefore, more objective evidence is needed to monitor intestinal function.

Gastrointestinal ultrasound [3] objectively measures the transverse area of the gastric antrum, the colon diameter and the frequency of peristalsis, so as to observe the gastrointestinal function. In this study, intra-abdominal pressure, gastric antrum ultrasonic cross-sectional area, left descending colonic/right descending colonic diameter, colonic peristatic frequency, EN start time, EN dose, prealbumin (PA), and EN complications were recorded. We dynamically monitored the process of enteral nutrition therapy, nourishing feeding, and targeted feeding, through statistical analysis, to evaluate function and determine the start time of enteral nutrition.

## Materials and methods

### Patients

This observational study was approved by the ethics committee of Zhangzhou Affiliated Hospital of Fujian Medical University (20180212D). Written informed consent to participate in the study was obtained from each patient.

The inclusion criteria for the research groups were as follows: > age of 18 years; AGI II; and hospitalization time greater than 120 h. The exclusion criteria were as follows: open abdominal trauma; abdominal tumor; large amount of peritoneal effusion; acute gastrointestinal function injury ≥ III magnitude; pregnancy.

We reviewed records of 105 patients admitted to the intensive care unit of Zhangzhou Hospital affiliated to Fujian Medical University from December 2018 to February 2020 who suffered acute gastrointestinal injury (AGI II) with NRS2002 score ≥ 3. After 6 h of stabilization, and vasoactive drugs begun to taper off, enteral nutritional suspension (SP, Short peptide based on whey protein hydrolysate, produced by Nutricia Pharmaceutical (Wuxi) Co., LTD, 1 kcal/ml) was administered. Nourishing feeding at 20 ml/H was given within 24 to 48 h after EN initiation. If there was no enteral nutritional intolerance, 40 ml/H of targeted feeding was given. We recorded 420 data points, the monitoring time points were before EN, 24 h EN, 72 h EN, and 120 h EN, including IAP, CSA, Diam, Peri, EN start time, EN dose, PA, and EN complications. The recovery of intestinal function after EN treatment was judged as success. Feeding intolerance occurs [4].When gastric residual volume (GRV) is 500 ml within 6 h and gastrointestinal symptoms such as vomiting, diarrhea, abdominal pain and distension occur,gastric motility drugs were allowed within 72 h of EN, and when the target feeding was not achieved after 120 h of EN, this was judged as failure.

### Intra Abdominal Pressure(IAP)

We represented intra abdominal pressure by measuring bladder pressure.The patient was placed in supine position with a catheter and the bladder emptied.The catheter was disinfected, then 100 mL NS was connected with a disposable syringe and 50 ml was injected into the bladder, then separated the disposable syringe.With the symphysis pubis as the zero point,connected the catheter to the ruler, released the drainage tube, and when the fluid level was stable, readed the value at the end of the patient's expiratory, which was the IAP.

### Ultrasound examination

The ultrasound machine we used was SonoSite Edge II (Copyright©2019 FUJIFILM SonoSite,Inc.All rights reserved.)

The subjects were examined using bedside antrum ultrasound and colonic ultrasound by skilled researchers with severe ultrasonic training certificates. During and after the examinations, we took care to avoid wound contamination and to disinfect the probes. We confirm that all methods were performed in accordance with the relevant guidelines and regulations.

#### Gastric antrum ultrasonography

**Fig. 1:**
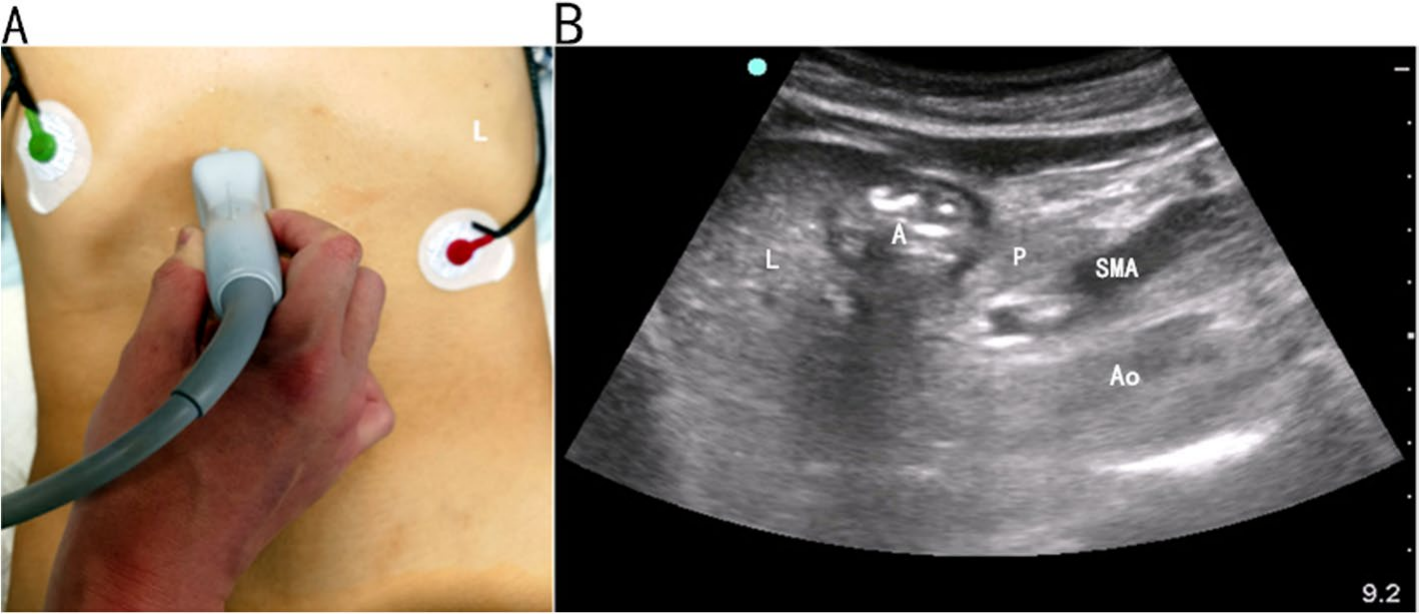
Ultrasonic examination of the gastric antrum. **A**:The Sonosite M-Tube to was used for monitoring, with the 2–5 MHz convex array probe. The patient lies on the right side (supine position for patients who cannot lie on the right side due to critical condition). The head of the bed is raised 30–45 degrees, and the direction of the probe is parallel to the longitudinal axis of the body, under the xiphoid process. Clear gastric antrum ultrasound was obtained at the right side of the median sagittal line [5]. **B**: Schematic diagram of standard section:L: liver. A: antrum of stomach. P: pancreas. SMA:superior mesenteric artery. Ao: aortaventralis

The ultrasonography was used for monitoring, with the 2–5 MHz convex array probe. The patient lies on the right side (supine position for patients who cannot lie on the right side due to critical condition). The head of the bed is raised 30–45 degrees, and the direction of the probe is parallel to the longitudinal axis of the body, under the xiphoid process. Clear gastric antrum ultrasound was obtained at the right side of the median sagittal line (Fig. 1 A/B) [5].

**Fig. 2:**
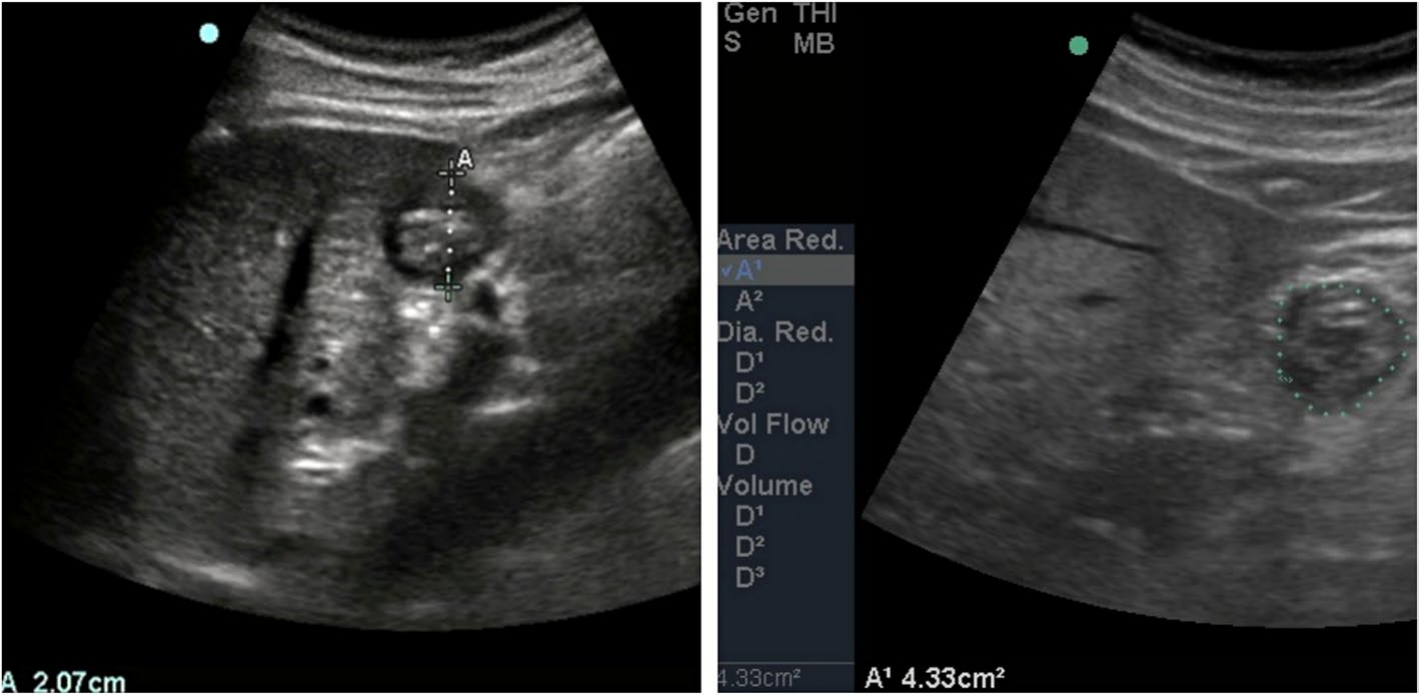
Scan and calculate the area of gastric antrum. Section of gastric antrum was taken by ultrasound, and then use Simpson 's integral method, trace along the edge of the gastric antrum, then utilizing ultrasonic data packet to scan and calculate the area of gastric antrum, we applied the following formula [6]: GRV (ml) = 27.0 + 14.6 × right-lateral CSA − 1.28 × age, to evaluate gastric volume

Section of gastric antrum was taken by ultrasound, and then use Simpson 's integral method, trace along the edge of the gastric antrum, then utilizing ultrasonic data packet to scan and calculate the area of gastric antrum [6], to evaluate gastric volume (Fig. 2).

#### Colonic ultrasonography

**Fig. 3:**
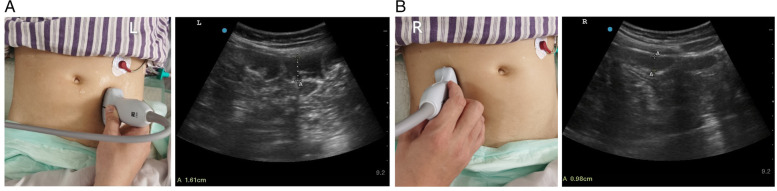
Left semicolon ultrasound and Right colonic ultrasound. **A**: The Sonosite M-Tube to was used for monitoring, with the 2–5 MHz convex array probe. The ultrasound probe was placed in the front line of the left axilla, and the direction of the probe was parallel to the longitudinal axis of the body, then obtained a clear image of the descending colon on the left. Then we measured the left descending colon diameter. **B**: The Sonosite M-Tube to was used for monitoring, with the 2–5 MHz convex array probe. The ultrasound probe was placed in the front line of the right axilla, and the direction of the probe was parallel to the longitudinal axis of the body, then obtained a clear image of the right ascending colon. Then we measured the inner diameter of the right ascending colon

We placed the 2–5 MHz convex array probe on the abdomen along the left descending colon or right descending colon, and scanned vertically from top to bottom. If necessary, the probe was rotated 90° to increase horizontal sliding scanning (Fig. 3A/B) [7]_._

### Test technical flow chart

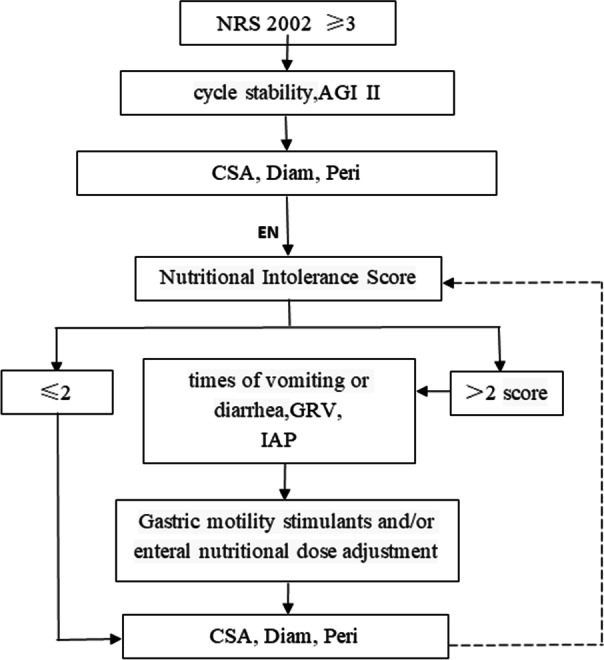



### Statistical analysis

#### Sample size

The incidence of major postoperative complications in a recent study with a similar definition was 1/3 [8].Considering this incidence rate, the variables used in sample size calculation are a ratio of sample sizes in negative/positive groups of 1, an area under the receiver-operating characteristics (ROC) curve of 0.7,a type I error probability (α) of 0.05, a type II error probability (β) of 0.1, and a null hypothesis value of 0.5. By using the MedCalc Statistical Software (Ver. 15.8, MedCalc Software Ltd., Ostend, Belgium), we calculated that 41 patients must be enrolled in each group (i.e., EN successful or EN failure). The minimum sample size was 82 patients. For the sake of data integrity, we included all patients in this time period, from December 2018 to February 2020, to anticipate a drop-out rate of 20% due to missing values. This study was retrospective and did not need to consider the rate of loss to follow-up [9].

Data were analyzed using SPSS version 20.0 software (IBM Corporation, Armonk, NY). All numeric data were expressed as mean ± standard deviation. Continuous variables between groups were compared using the independent samples t-test. We used MedCalc to draw receiver operating characteristic (ROC) curves. *P* < 0.05 was considered statistically significant.

## Results

A total of 105 patients were studied, and 420 data points were collected. There were 65 male patients and 40 female patients. The EN success group included 69 patients, and the failure group included 36 patients. The clinical characteristics of the study groups are shown in Table 1.

**Table 1:** Clinical characteristics of the two groups of patients

Characteristic	Successful (*n* = 69)	Failure (*n* = 36)	T	P
Age, y	71.84 ± 9.15	70.5 ± 5.9	-0.446	0.657
APACHE II	26.98 ± 1.43	27 ± 1.58	-0.048	0.962
IAP st, mmHg	17.78 ± 1.49	17.45 ± 1.96	0.958	0.34
Pa st, mg/L	69.39 ± 15.06	63.89 ± 15.03	1.793	0.076
Diseases, n (%)				
Pneumonia	11(15.9)	2(5.6)		
Heart failure	23(33.3)	12(33.3)		
Septic shock	19(27.5)	10(27.7)		
Celiac inflammation	8(11.6)	5(13.9)		
Pancreatitis	6(8.7)	2(5.6)		
MODS	2(3.0)	5(13.9)		

T-test was used for measurement data, and the ${\upchi }^{2}$ test was used for counting data. *P* < 0.05 was considered statistically significant

A total of 105 patients were studied, and 420 data points were collected. There were 65 male patients and 40 female patients. The EN success group included 69 patients, and the failure group included 36 patients. The clinical characteristics of the study groups are shown in Table 1

Single-factor comparison of EN initiation between the two groups. Analysis between the two groups showed that there were significant differences in terms of time of enteral nutrition initiation. When EN was started between the two groups, there were significant differences in CSA, Diam, PAand Peri, but no differences in IAP (Table 2).

**Table 2:** Single-factor comparison of EN initiation between the two groups

	EN		T	P
	Successful	Failure		
EN St	14.69 ± 8.98	19.51 ± 13.35	-2.21	0.029*
IAP	17.78 ± 1.46	17.46 ± 1.96	0.958	0.34
CSA	9.10 ± 1.32	10.75 ± 1.94	-5.18	< 0.001*
Diam	2.83 ± 0.31	3.37 ± 0.48	-7.1	< 0.001*
Peri	2.87 ± 0.98	2.29 ± 0.97	2.87	0.005*

Group 1 is the EN success group; Group 2 is the EN failure group. EN St, time to start enteral nutrition; IAP, intraperitoneal pressure, mmHg; CSA, the transverse antrum area of cm; Diam, diameter of left descending colonic or right ascending colonic, cm; Peri, peristalsis frequency, BPM; PA, prealbumin, mg/L.*P* < 0.05: have statistically significant

**Fig. 4:**
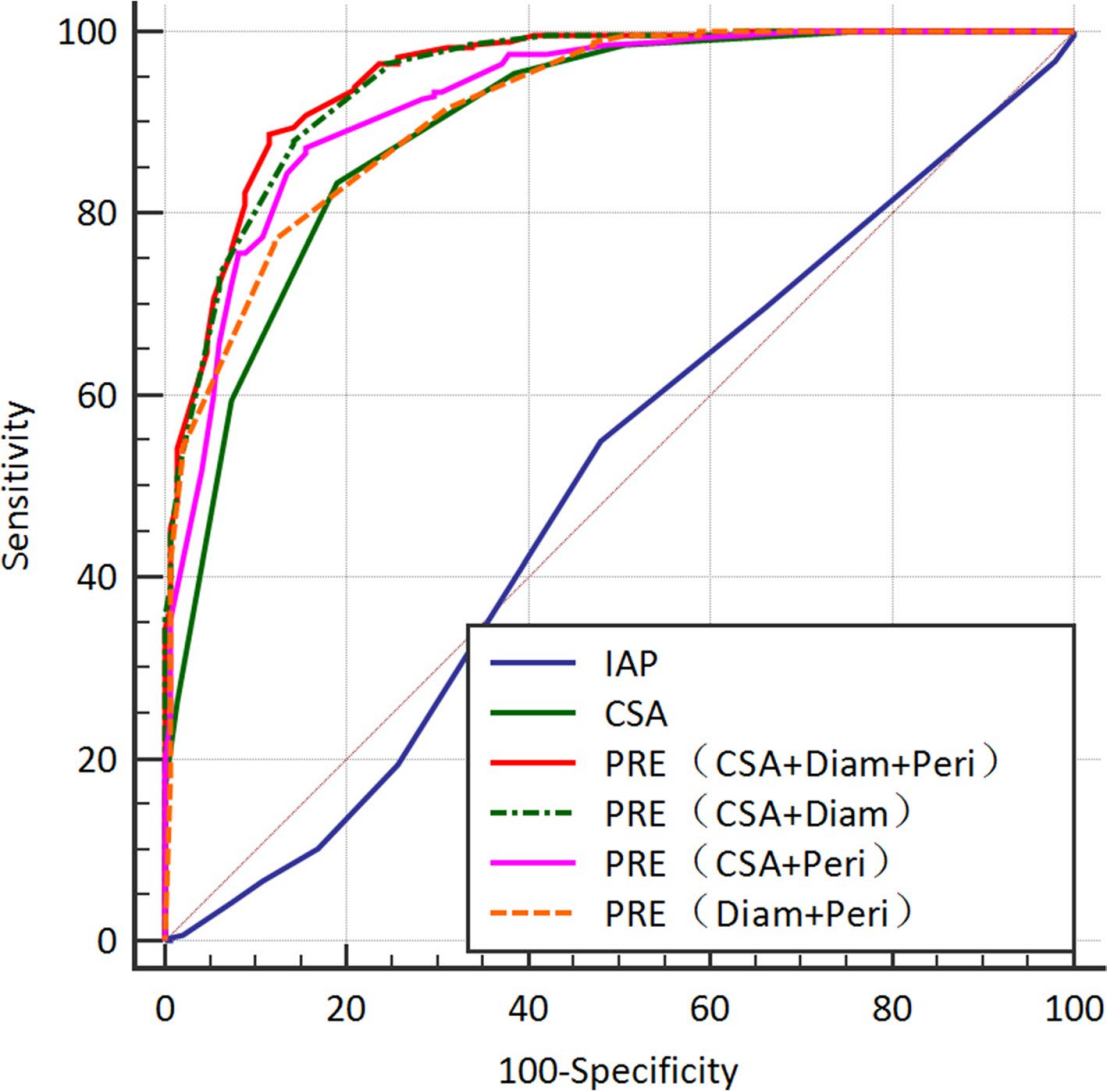
ROC curve of IAP, CSA, Diam, Peri,PRE. ROC curve of IAP, CSA, Diam, Peri and the joint evaluation of CSA, Diam, Peri: IAP has the lowest AUC area;CSA, Diam, Peri all have higher predicted values than IAP when compared with IAP.When CSA, D and F were combined to evaluate the success of EN, the positive predictive value was higher than that of single indicator.When CSA + Diam + Peri were combined evaluation, the area under the AUC curve was the largest and the positive predictive value was the highest.When Diam + Peri was used to evaluate the success of EN, there was no statistical difference with other indicators, the possible reason being that these two indicators could only represent colon function, not the recovery of the whole gastrointestinal function. *IAP* Intra-abdominal pressure, *CSA* Transverse area of gastric antrum, *Diam* left descending colonic or right ascending colonic diameter, *Peri* Peristatic frequency;PRE, the joint evaluation of transverse area of gastric antrum, colonic peristatic frequency and colonic diameter

ROC curve of IAP, CSA, Diam, Peri and the joint evaluation of CSA, Diam, Peri: IAP has the lowest AUC area;CSA, Diam, Peri all have higher predicted values than IAP when compared with IAP.When CSA, Diam and Peri were combined to evaluate the success of EN, the positive predictive value was higher than that of single indicator. When CSA + diameter + peristaltism evaluation were combined the area under the AUC curve was the largest and the positive predictive value was the highest. When diameter and peristaltism evaluation were combined to evaluate the success of EN, there was no statistical difference with other indicators, the possible reason being that these two indicators could only represent colon function, not the recovery of the whole gastrointestinal function (Tables 3 and 4, Fig. 4).

**Table 3:** IAP, CSA, Diam, Peri and PRE were used to evaluate gastrointestinal function recovery

	**cut–off**	**Youden index**	**Sensitivity 95% CI**	**Specificity 95% CI**	**AUC 95% CI**	**PPV 95% CI**	**NPV 95% CI**
IAP	≤ 16	0.07	54.93 (48.9—60.8)	52.03 (43.7–60.3)	0.502 (0.454–0.550)	68.7 (62.3—74.7)	37.6 (30.9—44.6)
CSA	≤ 9	0.65	83.45 (78.6–87.6)	81.08 (73.8–87)	0.896 (0.86–0.92)	89.4 (85.1–92.9)	71.9 (64.4–78)
Diam	≤ 2.9	0.6642	91.2 (87.3–94.2)	75.0 (67.2–81.7)	0.92(0.889–0.94)	87.5 (83.2–91.0)	81.6 (74.1–87.7)
Peri	> 3	0.566	70.77(65.1–76)	85.81(79.1–91)	0.845(0.808–0.878)	90.5(85.9–94)	60.5(53.5–67.1)
PRE (CSA + Diam + Peri)	> 0.68	0.77	88.73 (84.5—92.2)	88.51 (82.2—93.2)	0.95(0.925–0.97)	93.7 (90.1—96.3)	80.4 (73.4—86.2)
PRE (CSA + Diam)	> 0.58	0.74	88.03 (83.7—91.6)	85.81 (79.1—91.0)	0.946(0.92–0.96)	92.3 (88.4—95.1)	78.9 (71.8—84.9)
PRE (CSA + Peri)	> 0.55	0.72	87.32 (82.9—91.0)	84.46 (77.6—89.9)	0.925 (0.89–0.95)	91.5 (87.5—94.5)	77.6 (70.4—83.8)
PRE (Diam + Peri)	> 0.65	0.65	77.11 (71.8—81.9)	87.84 (81.5—92.6)	0.914 (0.88–0.94)	92.4 (88.3—95.4)	66.7 (59.6—73.2)

**Table 4:** Pairwise comparison of IAP, CSA, Diam, Peri and PRE

	**AUC difference**	**Standard error**	**95% CI**	**Z**	**P**
IAP-CSA	0.393	0.0297	0.335–0.452	13.252	< 0.001*
IAP-PRE(CSA, Diam**,** Peri)	0.448	0.289	0.391–0.504	15.523	< 0.001*
IAP-PRE(CSA, Diam)	0.444	0.029	0.387–0.501	15.286	< 0.001*
IAP-PRE(CSA, Peri)	0.423	0.0289	0.366–0.479	14.613	< 0.001*
IAP-PRE(Diam**,** Peri)	0.411	0.0301	0.352–0.470	13.679	< 0.001*
CSA-PRE(CSA, Diam**,** Peri)	0.0544	0.0113	0.032–0.076	4.832	< 0.001*
CSA-PRE(CSA, Diam)	0.0505	0.0108	0.0293–0.0718	4.658	< 0.001*
CSA-PRE(CSA, Peri)	0.0293	0.007	0.014–0.044	3.801	0.001*
CSA-PRE(Diam**,** Peri)	0.0179	0.017	-0.015–0.05	1.058	0.29
PRE(CSA, Diam**,** Peri)- PRE(CSA, Diam)	0.0038	0.002	-0.0004–0.008	1.742	0.08
PRE(CSA, Diam**,** Peri)- PRE(CSA, Peri)	0.025	0.006	0.0128–0.037	4.027	0.001*
PRE(CSA, Diam**,** Peri)- PRE(Diam**,** Peri)	0.036	0.008	0.018–0.054	4.07	< 0.001*
PRE(CSA, Diam)- PRE(CSA, Peri)	0.02	0.007	0.007–0.035	3.009	0.002*
PRE(CSA, Diam)- PRE(Diam**,** Peri)	0.03	0.01	0.0125–0.053	3.175	0.001*
PRE(CSA, Peri)- PRE(Diam**,** Peri)	0.011	0.011	-0.011–0.034	0.982	0.326

*IAP* Intra-abdominal pressure,mmHg, *CSA* Transverse area of gastric antrum,cm^2^, *Diam* Diameter of left descending colonic or right ascending colonic,cm, *Peri* Peristalsis frequency; *PRE* the joint evaluation of transverse area of gastric antrum and colonic diameter, colonic peristalsis frequency

ROC curve of IAP, CSA, Diam, Peri and PRE

6a:ROC curve of IAP, CSA, Diam**,** Peri and the joint evaluation of CSA, Diam**,** Peri: The sensitivity and specificity of different parameters were compared with the enteral nutrition success group: IAP ≤ 16 mmHg, AUC 0.502; CSA ≤ 9cm2, AUC 0.896; Diam ≤ 2.9 cm, AUC 0.92; Peri > 3 bpm, AUC0.845; CSA + Diam** + **Peri, AUC 0.95

6b: IAP, CSA, Diam**,** Peri and PRE, pairwise comparison showed statistical difference

## Discussion

The main fndings of our study were: 1. IAP did not accurately assess gastrointestinal functional status, nor could it guide the duration and the dose of EN therapy; 2. When EN was activated, there were statistical differences in the starting time, CSA, Diam and Peri of EN, suggesting that the successful group may had a better basis of gastrointestinal function and less gastrointestinal function injuried, which was conducive to the success of enteral nutrition therapy. 3. IAP, CSA, Diam and Peri were used as indicators to monitor gastrointestinal function recovery, and ROC curve was made. When comparing the single indicators of CSA, Diam and Peri with IAP, they all had higher predictive values than IAP. When the combination of CSA, Diam and Peri was used to evaluate the success of EN, the positive predictive value was higher than that of single indicator. When CSA + diameter + peristaltism evaluation were combined, the area under the AUC curve was the largest and the positive predictive value was the highest. 4. When Diam + Peri was used to evaluate the success of EN, there was no statistical difference between Diam + Peri and other indicators,the possible reason was that these two indicators only represented colon function, not the recovery of the whole gastrointestinal function.

The gastrointestinal tract has complex structure and many physiological functions. When there is an imbalance between systemic inflammatory response and compensatory anti-inflammatory response, injuries are often suffered [10]. In the study of 242 patients with mechanical ventilation for more than 48 h, the incidence of gastrointestinal bleeding was 46.7% [11]. Appropriate mechanical ventilation Settings can protect gastrointestinal function [12, 13]. High-pressure ventilator used, renal failure, and reduced platelet count are risk factors for gastrointestinal bleeding, while enteral nutrition was an independent protective factor [14]. In our study, no patients experienced bleeding or other complications as a result of enteral nutrition therapy, and the most common nutritional intolerance was still gastric retention. The current gastric retention rate is 6 h and GRV > 500 ml, the patient is prone to vomiting, aspiration and other risks, whether it is necessary to redefine the judgment criteria of gastric retention, earlier implementation of gastric motility and other intervention measures.

Successful enteral nutrition therapy in critically ill patients can promote gastrointestinal mucosal repair, promote gastrointestinal function recovery, and reduce endogenous infection [15]. However, some patients have feeding intolerance, including gastric retention, diarrhea and abdominal distension [16]. Therefore, an effective and comprehensive assessment of gastrointestinal functional status is very important to guide the timing of enteral nutrition therapy initiation [17]. and the amount of feeding [18].

At present, enteral nutrition is recommended to be started 24–48 h after the stabilization of the disease. By monitoring gastrointestinal function indicators such as gastric antrum cross-section area, colon diameter and peristalsis frequency [19]. Enteral nutrition is recommended to be started 6 h after the stabilization of the disease, when the amount of vasoactive drugs is gradually reduced. There were statistical differences in ENst, CSA, Diam and Peri in the EN successful group, suggesting that gastrointestinal function may have begun to recover 6 h after disease control, which can determine whether the improvement of gastrointestinal function and the start of enteral nutrition can be earlier.

In previous studies of our team, it was found that intra-abdominal pressure could not reflect the absorption function of intestinal function. In IAP similar states, although circulation is stable, some patients do not have synchronous recovery of intestinal function [8]. This phenomenon is not reflected by abdominal examination and IAP [20]. It indicates that gastrointestinal ultrasound can better evaluate the damage state of gastrointestinal function, and enteral nutrition therapy can be carried out earlier [21].

Continuous feeding of short peptide nutritional preparations can achieve the target calorie [22]. There were fewer feeding complications. In our study, whey protein hydrolysates were also used for feeding. When enteral nutrition is absorbed, it promotes intestinal mucosal repair and contributes to the recovery of humoral and cellular immune function in the early post-traumatic period [23].

Our study found that when IAP was evaluated for gastrointestinal function, it was 17.78 ± 1.46 mmHg in the success group and 17.46 ± 1.96 mmHg in the failure group, *P* > 0.05, there was no statistical difference between the two groups; When ROC was used to determine the IAP value of the successful group, AUC of ROC 0.502, and the correlation analysis between IAP and PA, *R* = 0.139 [8], suggested that IAP could not accurately assess gastrointestinal function.

Since there was no relevant literature on the combined assessment of gastrointestinal function by CSA, Diam, Peri and other indicators; Our study found that CSA ≤ 9cm^2^, AUC 0.896; Diam ≤ 2.9 cm, AUC 0.92; Peri > 3 bpm, AUC 0.845; The results indicated that the three indexes could reflect the recovery of gastrointestinal function. When CSA, Diam and Peri were combined, the positive predictive value and AUC were higher than those of single indicator, and the difference was statistically significant (*P* < 0.001), except Diam + Peri combination (*P* > 0.05), indicating that only Diam and Peri could not reflect the complete gastrointestinal function. Patients with CSA ≤ 9cm^2^, D ≤ 2.9 cm, Peri > 3 bpm, enteral nutrition therapy was initiated with an AUC of 0.95 and the success rate of enteral nutrition therapy was 93.7%, suggesting that enteral nutrition therapy could be initiated earlier without complications after a more complete evaluation of gastrointestinal function.

The disadvantage of this study is that the single center study has a small number of cases. Since the site monitored in this test is the diameter of the colon, and most of the nutrient absorption is carried out in the small intestine, it needs to be further confirmed whether we can use ultrasound to further monitor the function of the small intestine in the future.

## Conclusions

When we use ultrasound to monitor CSA, Diam and Peri for joint evaluation, we can comprehensively and objectively reflect the functional impairment and recovery of gastrointestinal tract, predict the success rate of enteral nutrition therapy, and guide the time to start enteral nutrition therapy.

## Supplementary Information

**Additional file 1. **Research data.

## Data Availability

All data generated or analysed during this study are included in this published article [and its supplementary information files].

## Abbreviations

AGI: Acute gastrointestinal injury
APACHE II: Acute Physiology and Chronic Health Evaluation II
CARS: Compensatory Anti-inflammatory Response Syndrome
CSA: Transverse area of gastric antrum
Diam: Left descending colonic or right ascending colonic diameter
EN: Enteral nutrition
Peri: Peristatic frequency
IAP: Intra-abdominal pressure
SIRS: Systemic Inflammatory Response Syndrome
PA: Prealbumin
PRE: The joint evaluation of transverse area of gastric antrum and colonic diameter

## References

[1] Reintam Blaser A, Starkopf J, Alhazzani W, Berger MM, Casaer MP, Deane AM (2017). Early enteral nutrition in critically ill patients: ESICM clinical practice guidelines. Intensive Care Med.

[2] Singer P, Reintam Blaser A, Berger MM, Alhazzani W, Calder PC, Casaer M (2019). ESPEN guideline on clinical nutrition in the intensive care unit. Clin Nutr.

[3] Yin MG, Wang XT, Liu DW (2018). Technical specification for clinical application of critical ultrasonography. Zhonghua Nei Ke Za Zhi.

[4] Jones NE, Dhaliwal R, Day AG (2008). Factors predicting adherence to the Canadian Clinical Practice Guidelines for nutrition support in mechanically ventilated, critically ill adult patients. J Crit Care.

[5] Wang XT, Liu DW, Yu KJ, et al. Experts’ opinions on critical ultrasonography in China. Zhonghua nei ke za zhi. 2016;55:900–12. 10.3760/cma.j.issn.0578-1426.2016.11.020. 10.3760/cma.j.issn.0578-1426.2016.11.020 27801352

[6] Perlas A, Mitsakakis N, Liu L (2013). Validation of a mathematical model for ultrasound assessment of gastric volume by gastroscopic examination. Anesth Analg.

[7] Bouvet L, Chassard D (2013). Ultrasound assessment of gastric volume: what is the best threshold?. Anesth Analg..

[8] Zheng QJ, Lai JW, Chen LL (2021). The value of enteral nutrition in critically ill patients evaluated by gastrointestinal ultrasound combined with AGI. Chin J Emerg Med..

[9] Zhou XF, Yu RG, Chen H. et al. Performance of Lactate and CO2-Derived Parameters in Predicting Major Postoperative Complications After Cardiac Surgery With Cardiopulmonary Bypass:Protocol of a Diagnostic Accuracy Study. Front Cardiovasc Med. 8:724713. 10.3389/fcvm.2021.724713. 10.3389/fcvm.2021.724713 PMC8517114 34660725

[10] Heyland DK, Dhaliwal R, Drover JW, Gramlich L, Dodek P (2003). Canadian clinical practice guidelines for nutrition support in mechanically ventilated, critically ill adult patients. JPEN J Parenter Enteral Nutr.

[11] ReintamBlaser A, Malbrain ML, Starkopf J, et al. Gastrointestinal function in intensive care patients: terminology, definitions and management. Recommendations of the ESICM Working Group on Abdominal Problems. Intensive Care Medicine. 2012;38:384–94. 10.1007/s00134-011-2459-y. 10.1007/s00134-011-2459-y PMC3286505 22310869

[12] Chen H, Xu M, Yang YL (2017). Application of injection test in confirming the ideal position of esophageal balloon catheter. Chin Crit Care Med.

[13] Chen H, Yang YL, Xu M (2017). Use of the injection test to indicate the oesophageal balloon position in patients without spontaneous breathing: a clinical feasibility study. J Int Med Res.

[14] Nigeen J and Timothy G: Recent Advances in Managing Acute Pancreatitis. F1000research. 2015. 10.12688/f1000research.7172.1. 10.12688/f1000research.7172.1 PMC4754010 26918139

[15] Yao MQ, Feng XQ, Guo ZT (2018). Early enteral nutrition support dose selection in critically ill patients: a Meta analysis. Chin J Emerg Med.

[16] Mcclave SA, Dibaise JK, Mullin GE, Martindale RG (2016). ACG Clinical Guideline: Nutrition Therapy in the Adult Hospitalized Patient. Am J Gastroenterol.

[17] Shukla A, Chapman M, Patel JJ (2021). Enteral nutrition in circulatory shock: friend or foe?. Curr Opin Clin Nutr Metab Care..

[18] Andrea P, Balázs K, Andrea S (2016). Prospective, Multicentre, Nationwide Clinical Data from 600 Cases of Acute Pancreatitis. Plos One..

[19] Jiang Y, Hu B, Zhang S (2020). Effects of early enteral nutrition on the prognosis of patients with sepsis: secondary analysis of acute gastrointestinal injury study. Ann Palliat Med..

[20] Deane AM, Ali Y, Plummer MP (2020). Are Classic Bedside Exam Findings Required to Initiate Enteral Nutrition in Critically Ill Patients: Emphasis on Bowel Sounds and Abdominal Distension. Nutr Clin Pract.

[21] Li Y, Yang J, Sun S (2020). Effects of intermittent feeding versus continuous feeding on enteral nutrition tolerance in critically ill patients: A protocol for systematic review and meta-analysis. Medicine (Baltimore)..

[22] Hrdy O, Vrbica K, Strazevska E (2020). Comparison of continuous versus intermittent enteral nutrition in critically ill patients (COINN): study protocol for a randomized comparative effectiveness trial. Trials.

[23] Sun H, Yang Q, Ke Li (2020). Effect of different methods of applying enteral nutritional support methods in elderly patients with severe pneumonia. Chin J Emerg Med..

